# Challenging the negativity bias in affective scene viewing: The role of social content

**DOI:** 10.1093/scan/nsaf108

**Published:** 2025-10-14

**Authors:** Anna Fischer, Danilo Postin, Lina Johanna Meiners, Louisa Kulke, Pascal Vrtička, Anne Schacht

**Affiliations:** Department of Cognition, Emotion and Behavior, Institute of Psychology, Georg August University of Goettingen, Goettingen, Germany; Department of Cognition, Emotion and Behavior, Institute of Psychology, Georg August University of Goettingen, Goettingen, Germany; Department of Psychiatry, School of Medicine and Health Sciences, Carl von Ossietzky University Oldenburg, Oldenburg, Germany; Department of Cognition, Emotion and Behavior, Institute of Psychology, Georg August University of Goettingen, Goettingen, Germany; Department of Cognition, Emotion and Behavior, Institute of Psychology, Georg August University of Goettingen, Goettingen, Germany; Department of Developmental Psychology with Educational Psychology, University of Bremen, Bremen, Germany; Centre for Brain Science, Department of Psychology, University of Essex, Colchester, UK; Department of Cognition, Emotion and Behavior, Institute of Psychology, Georg August University of Goettingen, Goettingen, Germany

**Keywords:** social content, emotional valence, negativity bias, event-related potentials, eye tracking

## Abstract

How does the brain prioritize information when visual scenes contain multiple sources of relevance? While emotionally evocative content has long been considered central to attentional capture, social content constitutes another key dimension of relevance. However, the neural mechanisms underlying the integration of these relevance dimensions remain unclear. We co-registered event-related potentials (ERPs) and eye movements while participants viewed complex scenes varying in social content (social, non-social) and emotional valence (positive, negative, neutral). Early ERP responses (P1) showed enhanced amplitudes for positive social images, suggesting that social relevance can mitigate the early-stage negativity bias. Social content also modulated the EPN, while emotional valence shaped later components (P300, LPC), with larger amplitudes for negative scenes. Eye-tracking measures mirrored these ERP effects: initial saccades were faster for social images, and fixation patterns indicated increased visual exploration for both positive and negative social scenes relative to neutral ones. Together, these results support a sequential appraisal process in which social content is prioritized at early perceptual stages, while emotional valence influences later evaluative processing. This pattern challenges the notion of a general negativity bias and underscores the interactive and stage-specific contributions of social and emotional relevance in affective scene perception.

## Introduction

Our brains continuously prioritize information most relevant for adaptive behaviour in complex environments. While emotional cues have long been emphasized, accumulating evidence also highlights the crucial role of social content and its influence on emotional appraisal (Hariri *et al.* 2002; Mumenthaler and Sander 2012; Feng *et al.* 2021). Yet the precise neural mechanisms underlying rapid attention to emotionally and socially relevant stimuli remain unclear, and it is debated whether these dimensions are integrated at early stages or appraised independently (Sander *et al.* 2005; Moors *et al.* 2013). This uncertainty may stem from experimental designs in which emotional and social relevance are confounded within the same stimuli. In much previous work (e.g. Bradley *et al.* 2001; Schupp et al. 2007), socially relevant cues such as human faces inherently carried emotional meaning (e.g. smiling or fearful expressions), making their individual contributions difficult to disentangle.

Emotional stimuli are prioritized in perception, detected more rapidly, and reach consciousness more readily than neutral stimuli (Brosch *et al.* 2010). This prioritization is typically stronger for negative content, reflecting a ‘negativity bias’ (Cacioppo *et al.* 1999; Öhman and Mineka 2001; Crawford and Cacioppo 2002), but can also occur for positive stimuli with high motivational relevance (cf Pool *et al.* 2016). Because positive scenes often involve people, emotional and social content are frequently confounded. Studies on complex scenes show that social features capture attention automatically, even in the presence of other salient elements (End and Gamer 2017; Rösler *et al.* 2017; Flechsenhar *et al.* 2018; Skripkauskaite *et al.* 2023), yet few studies have disentangled their specific contributions.

Electroencephalography (EEG) and eye tracking provide complementary, high-temporal-resolution measures of relevance processing: eye tracking captures gaze behaviour, and EEG reveals neural dynamics over time (Nikolaev *et al.* 2016; Zheng *et al.* 2019). Several emotion-sensitive event-related potentials (ERPs) reflect distinct processing stages. The P1 component, originating in occipital areas, indexes early sensory processing and attentional selection (Luck *et al.* 2000) and can be modulated by emotional content (Rellecke *et al.* 2011; Mielke *et al.* 2022). The early posterior negativity (EPN), linked to motivational attention, is enhanced for emotionally arousing stimuli (Schupp *et al.* 2004; Junghöfer et al. 2010) and sensitive to human bodies (Frank and Sabatinelli 2019; Farkas *et al.* 2020). The P300 and the Late Positive Component (LPC) are associated with motivational relevance, task engagement, and sustained attention (Schupp *et al.* 2006; 2007; Mastria *et al.* 2017).

Appraisal theories of emotion propose that affective responses result from successive evaluations of a stimulus’s significance for the individual (Moors *et al.* 2013). Within this framework, the Component Process Model (CPM; Scherer 2001) specifies a sequence of appraisal checks assessing novelty, intrinsic pleasantness, goal conduciveness, and coping potential, which together determine the motivational relevance of a stimulus. Subsequent theoretical extensions have highlighted that social content constitutes a particularly important source of relevance, as it directly informs evaluations of safety, affiliation, and norm compatibility (Sander *et al.* 2005; Mumenthaler and Sander 2012). Social cues may therefore shape early appraisal outcomes and bias attention already at initial perceptual stages. The CPM’s temporally structured architecture aligns particularly well with the millisecond precision of ERP measures, allowing theoretical mapping between appraisal stages and electrophysiological components. Accordingly, early components such as the P1 may reflect novelty and intrinsic pleasantness appraisals—potentially modulated by the intrinsic relevance of social cues—whereas the EPN may index relevance detection and motivational significance. Later components, such as the P300 and LPC, may correspond to higher-order evaluations and memory-related integration. Eye-tracking measures such as saccade latency, amplitude, and fixation patterns provide behavioural correlates of these stages, capturing overt attention deployment and information sampling that accompany neural processing dynamics.

Affective picture processing has been widely studied with ERP (Olofsson *et al.* 2008) and eye-tracking methods, but few studies have treated social content as a complementary stimulus dimension (Proverbio *et al.* 2009; Okruszek *et al.* 2016; End and Gamer 2017; Schacht and Vrtička 2018). These studies show that depictions of people or faces can modulate emotion-sensitive ERPs, including the P1, EPN, and LPC. Okruszek et al. (2016) found early ERP effects of social content in negative and neutral scenes, and when positive scenes were included, P1 amplitudes were larger for positive than negative social images (Proverbio *et al.* 2009; Schacht and Vrtička 2018). These findings suggest that the increased social relevance of positive stimuli can attenuate the typical negativity bias (e.g. Bayer and Schacht 2014), indicating that emotional and social content already interact at early perceptual stages (Schacht and Vrtička 2018).

Eye tracking provides valuable insight into the temporal dynamics of relevance processing, yet few studies have examined the joint effects of emotional and social content during natural scene viewing. Considered separately, both factors shape gaze behaviour during picture viewing: emotional valence influences eye movements (Bradley *et al.* 2011; Humphrey *et al.* 2012; Simola *et al.* 2015), and emotionally evaluated regions—rather than physically salient ones—better predict gaze patterns (Niu *et al.* 2012). Social features also strongly predict gaze behaviour, even beyond low-level physical salience, and this prioritization appears partly automatic (Birmingham *et al.* 2009; End and Gamer 2017; Rösler *et al.* 2017).

The present study examined the temporal dynamics of processing social content and emotional valence in complex visual scenes. Social content was defined as the visible presence of at least one human figure, typically embedded in an implied or overt interpersonal context. Most social stimuli depicted full-body scenes; four close-up portraits were included in the neutral condition for balance. Unlike traditional emotional-face paradigms, which isolate expressions from context, our scenes conveyed social meaning through contextual, bodily, and situational cues, and even single-person images implied interaction. A companion rating study (Mitschke *et al.* 2025) confirmed that perceived social relevance is not reducible to the number of visible faces or people. To address confounds identified in prior work (Tatler 2007; Birmingham *et al.* 2009; Tseng *et al.* 2009; Vincent *et al.* 2009; Bradley *et al.* 2011; End and Gamer 2017; Durmus 2020), we employed a fully orthogonal design (social vs. non-social × positive, negative, neutral), controlled for low-level features, and verified image complexity, physical salience, and spatial distribution of relevant regions. This approach avoids the conflation of social relevance with emotional meaning that occurs, for example, when using emotionally expressive human faces as stimuli, thereby allowing us to examine their independent and interactive contributions.

Building on Schacht and Vrtička (2018), we co-registered EEG and eye tracking to examine attentional engagement at both neural and behavioural levels, using a larger and more strictly controlled image set, and applied generalized linear mixed models (GLMMs) with crossed random effects (Baayen et al. 2008) to model trial-by-trial variability. This approach is grounded in appraisal theories, which posit a sequence of relevance checks (Scherer 2001; Sander *et al.* 2005). Within this framework, and based on previous findings (Proverbio *et al.* 2009; Schacht and Vrtička 2018), we hypothesized an early interaction between social and emotional relevance in P1 amplitudes. For later ERP components (EPN, P300, LPC), we expected either interactive or dissociable mechanisms, reflecting integrated versus sequential relevance processing.

We also anticipated systematic modulations of gaze behaviour, as both social (Birmingham *et al.* 2009; Parks *et al.* 2015; End and Gamer 2017; Rösler *et al.* 2017) and emotional (Humphrey *et al.* 2012; Pilarczyk and Kuniecki 2014) relevance enhance attentional engagement. Specifically, we expected shorter saccade latencies and larger saccade amplitudes for socially and emotionally salient scenes, reflecting rapid orienting and broader exploration, as well as more fixations of shorter duration, indicating sustained information seeking. Conceptually, these eye-movement indices parallel the temporal cascade observed in ERP components: saccade latency (attention capture; Rösler *et al.* 2017; Skripkauskaite *et al.* 2023), and saccade amplitude (scan path extent; Nummenmaa *et al.* 2009; Bradley *et al.* 2011; Simola *et al.* 2013; Simola *et al.* 2015; End and Gamer 2017; Rösler *et al.* 2017; Skaramagkas *et al.* 2023), correspond to early orienting reflected in the P1 and EPN, whereas the number and duration of fixations (information seeking; Bradley 2009; Bradley et al. 2011) align with later evaluative stages captured by the P300 and LPC.

## Methods

The study design, research questions, and hypotheses were preregistered in the Open Science Framework (OSF; https://osf.io/vxn6g; https://osf.io/4wrzf) as part of two undergraduate theses. Ethical approval was obtained from the Institute of Psychology, University of Goettingen, Germany (application number: 70).

### Participants

Twenty-seven participants were initially recruited. Inclusion criteria were: healthy status; right-handedness (assessed via the Edinburgh Handedness Inventory; Oldfield 1971); age between 18 and 35 years; and normal or corrected-to-normal vision. Only female participants were included to control for potential sex differences in EEG responses to emotional stimuli (Proverbio *et al.* 2009; Kato and Takeda 2017) and eye-movement behaviour (Mercer Moss *et al.* 2012). Three participants were excluded due to EEG or eye-tracking artifacts, yielding a final sample of 24 healthy female students (age range = 19–28 years, M = 21.71, SD = 2.20). The sample size was determined by participant availability for two coordinated master’s theses and is comparable to previous ERP studies on emotional scene perception. Participation was voluntary and compensated with course credit.

### Apparatus

Stimuli were presented at the centre of a 24’’ monitor (1920 × 1080 px, 144 Hz refresh rate) at a viewing distance of 76 cm. Images (512 × 384 px) subtended a visual angle of 10.6° × 8.0°. Stimulus presentation was controlled using Presentation software (Neurobehavioral Systems, Berkeley, USA), which also triggered synchronization pulses to the EEG and eye-tracking systems. Eye movements were recorded with an EyeLink^®^ 1000 eye-tracker (SR Research, Kanata, Canada). Participants’ heads were stabilized using a chin rest, and the position of their right eye was recorded at a 1000 Hz sampling rate. The pupil was tracked using the ellipse model. A nine-point calibration routine was used in randomized order, and calibration was accepted if the average accuracy error was below 0.5° (M = 0.35°, SD = 0.08°).

EEG was recorded from 64 electrodes using an Easy-Cap (BioSemi, Amsterdam, The Netherlands), with electrode impedance kept below 30 kΩ. Data were sampled at 512 Hz with a bandwidth of 104 Hz. Common mode sense (CMS) and driven right leg (DRL) electrodes served as reference and ground, respectively. Electrooculogram (EOG) was recorded from external electrodes placed lateral to and below the eyes, and signals from left and right mastoids were additionally recorded.

### Stimulus material

Pictures were selected from the International Affective Picture System (IAPS) database (Bradley *et al.* 2007) and online sources. They depicted complex visual scenes that varied in social content (social, non-social) and emotional valence (positive, negative, neutral), resulting in six experimental conditions. All neutral pictures were taken from the IAPS (neutral social: 2190, 2191, 2200, 2214, 2215, 2381, 2383, 2393, 2480, 2487, 2495, 2514, 2516, 2595, 2635, 2749, 2840, 2850, 2890, 7640; neutral non-social: 1935, 5531, 5534, 5535, 6150, 7002, 7034, 7035, 7036, 7037, 7038, 7041, 7050, 7090, 7161, 7170, 7211, 7217, 7233, 7235), and some of the positive (social: 2057, 2340, 4700; non-social: 1440, 5626) and negative pictures (social: 2141, 2900, 9041, 6821, 6834). Each positive and negative condition included 40 pictures (e.g. 40 social positive, 40 non-social positive), while each neutral condition included 20 images. Social scenes featured clear interpersonal interactions, such as fighting, grief or loss, or other threat-related contexts (negative), and playing together, affiliative interactions, or recreational activities (positive). Non-social scenes lacked direct human presence, depicting, for example, a dead bird in industrial waste or an emotionally intense accident (negative), and a tropical island or other uplifting environments (positive). Images were matched for low-level visual features and validated in a pre-study rating. While stylistic differences between IAPS and non-IAPS sources cannot be fully excluded, no systematic differences were found in the rated dimensions. Social and non-social images were matched on pleasantness and arousal; detailed descriptions of the pre-experimental ratings for the full image set are provided in Vrtička et al. (2011, 2012).

Stimuli were matched for social content and emotional valence across the six conditions. Pre-experimental ratings of pleasantness and arousal confirmed that social and non-social images were equivalent in emotional arousal within each valence category. Full validation procedures and statistical results are provided in the Supplementary Material. Because definitions of ‘social’ versus ‘non-social’ content vary across studies, we further characterized our stimuli by having three independent raters count the number of visible people and faces in each image. These counts are reported in Supplementary Table S1. Due to limited statistical power, we did not model these counts but acknowledge the imbalance as a limitation (see Discussion). Most neutral images depicted a single clearly visible person or face, while most positive and about half of the negative images included two or more people. A follow-up behavioural study further assessed whether participants’ relevance judgments differed across conditions. Ratings confirmed the intended manipulation of social content and emotional valence (see Supplementary Material for detailed methods and statistical results).

In addition, 60 scrambled images were presented as distractors. They were randomly drawn from a pool of 120 scrambled versions of the intact pictures, each created by dividing the original image into 3,072 randomly distributed small squares (Schacht and Vrtička 2018).

All images were presented in full colour.

### Experimental procedure

Before the test session, participants provided written informed consent and demographic information. After EEG preparation, eye-tracker calibration was performed. The experiment included four blocks of ∼10 minutes each, with self-paced breaks in between. Each block consisted of 10 intact images per condition and 15 scrambled distractors presented in pseudo-randomized order, yielding 75 trials per block and 300 trials in total. Neutral images were presented twice to balance the trial counts.

Each trial began with a fixation cross 4,000 ± 2,000 ms (jittered), followed by the image (2,000 ms) and a question mark (2,000 ms), indicating the response window. Participants responded by button press on every trial, indicating whether the image was intact or scrambled, using the right hand; key assignments (left/right arrow) were counterbalanced. If a response occurred during image presentation, participants received ‘Too early!’ feedback, indicating that the trial would not count toward the main task performance. All stimuli and response cues were centrally presented on a black background, with response cues (fixation cross, feedback, question mark) displayed in white.

### Data pre-processing

EEG data were processed using Matlab (R2019, MathWorks Inc.) and the EEGLAB toolbox (Version 13_6_5b; Delorme and Makeig 2004). Bad channels were identified and interpolated globally per participant; only three datasets required interpolation, each affecting a maximum of two lateral temporal channels. EEG data were downsampled to 500 Hz and filtered offline using a Butterworth band-pass filter (0.5–40 Hz, 12 dB/oct). Signals were re-referenced to the average of all electrodes. A consistent 10-ms delay between stimulus onset and event marker registration was identified during pilot testing using photodiode measurements, which showed a systematic discrepancy between trigger timestamps and the actual screen refresh onset. Based on these measurements, a uniform 10-ms correction was applied to all stimulus triggers during preprocessing. The continuous EEG signal was segmented into 1200 ms epochs (–200 to +1000 ms relative to stimulus onset) and baseline corrected using the 200 ms pre-stimulus interval. Epochs with voltage exceeding ± 500 μV or with excessive eye disturbance (see eye-tracking pre-processing) were rejected. Outliers were further excluded using joint probability measures in EEGLAB (Delorme *et al.* 2007), with participant-specific thresholds gradually adjusted to limit rejection to a maximum of 10% of epochs per participant.

Following artifact rejection, independent component analysis (ICA) was performed using the AMICA algorithm in EEGLAB (Leutheuser *et al.* 2013). Only independent components (ICs) clearly reflecting eye movements were excluded. IC rejection was based on visual inspection of their scalp topographies and power spectra, as well as characteristic blink (vertical) or step-function (horizontal) activity. After preprocessing, an average of 90% of trials per participant was retained. Trials with premature responses were excluded from all ERP and eye-tracking analyses to ensure valid viewing intervals; these accounted for approximately 1.9% of trials per participant (*M *= 5.75 out of 300, *SD *= 4.22).

ERPs were quantified in line with prior work (Schacht and Vrtička 2018) to ensure comparability. P1 mean amplitudes were extracted 100–140 ms post-stimulus at PO7 and PO8. Mean amplitudes for the following EPN, P300, and LPC were extracted from a posterior electrode cluster (P9, PO7, O1, Iz, Oz, O2, PO8, and P10) in the time windows 200–320 ms (EPN), 320–420 ms (P300), and 420–620 ms (LPC), respectively. This shared-ROI approach follows prior work to facilitate comparability but may obscure component-specific topographies. To address this, we conducted complementary mass univariate analyses (MUAs) across a broader set of posterior electrodes and time points (see Supplementary Figures S2–S4), which confirmed the component-specific scalp distributions. The limitations of the shared-ROI approach are acknowledged in the Discussion.

**Figure 1.**
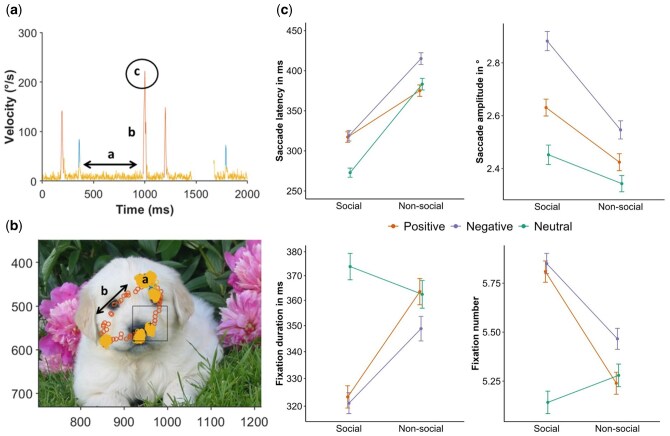
Example trial illustrated by eye movement Velocity (°/s) plotted over Time (a). Gaze plot with eye-position samples plotted on the presented stimulus (512 × 384 pixels; b). Within both plots, yellow data points represent fixations and orange points represent saccades which were detected by an adaptive saccade and fixation detection algorithm. The gap around 1500 ms results from data removed due to the occurrence of blinks. Ocular event (a, in panel a) represents a fixation with the arrow indicating the fixation duration and (b, in panel a) illustrates a saccade characterized by high Velocity rise and fall in (a) and distance travelled in (b). In (c) the Velocity peak (∼230°/s) of the second saccade detected in this trial is marked. The grand average means and standard errors for the eye movement data are shown on the right (c; error bars denote ± 1 standard error of the mean).

Eye-tracking data were preprocessed using Matlab (R2019, MathWorks Inc.). Blinks were detected using a spatial-based algorithm applied to raw eye position samples and replaced with missing values for at least 100 ms to eliminate residual artifacts. The raw data were then smoothed using a Savitzky-Golay low-pass filter (order = 2, window length = 10 ms; Nyström and Holmqvist 2010), and velocity and acceleration were computed from the filtered signal. Samples exceeding a velocity of 750°/s were replaced with missing values (Duchowski 2017). Trials with more than 50% missing values were discarded, ensuring at least 1 s of valid data per trial. Saccades and fixations were identified using adaptive threshold algorithms that account for individual noise levels and outperform fixed-threshold methods. Saccades were detected based on velocity and acceleration criteria, and their onset and offset were determined using the algorithm described by Nyström and Holmqvist (2010). Fixations were defined in the intervals between saccades (see Figure 1a, b for illustration). Eye-position samples not classified as saccade or fixation were labeled as undefined noise.

Eye-movement metrics were selected based on previous studies demonstrating attentional sensitivity to social and emotional content (Nummenmaa *et al.* 2009; Bradley *et al.* 2011; Simola *et al.* 2013; 2015; End and Gamer 2017; Rösler *et al.* 2017; Skaramagkas *et al.* 2023). From the detected saccades and fixations, we extracted four metrics per trial: (1) number of fixations, (2) average fixation duration, (3) average saccade amplitude (distance between onset and offset), and (4) saccade latency (time to first saccade onset).

### Statistical analysis

#### Behavioural data

Behavioural data were analysed descriptively to assess task compliance and response accuracy. We computed means (*M*) and standard deviations (*SD*) for the number of accurate and too-early responses to scrambled and intact images.

#### ERPs and eye-movement metrics

ERPs and eye-movement metrics were analysed using Generalized Linear Mixed Models (GLMM; Baayen *et al.* 2008) on single-trial data. GLMMs were used instead of the pre-registered ANOVA models to avoid violating prior assumptions (e.g. non-normal distribution of response variables), to include subject and image ID as random effects in all models, and thus to draw more meaningful conclusions. Fixed effects included social content (social, non-social), emotional valence (neutral, positive, negative), and their interaction. Random intercepts were included for both subject and image ID. To keep type I error rate at the nominal level of 0.05, we included random slopes (Schielzeth and Forstmeier 2009; Barr *et al.* 2013) for all fixed effects and their interaction in the random effect subject ID. Each model was fitted with a maximal random slope structure, ie, including correlations between intercepts and slopes. Emotional valence was dummy coded with neutral as the reference category, and both predictors were mean-centred before entering as random slopes. Prior to model fitting, the Variance Inflation Factors (VIF; Field 2005) were calculated from a model excluding the interaction term and indicated no collinearity (VIF = 1.000 for both predictors). The models were fitted in R Studio (version 2023.03.0.; R version 4.2.3) using the package lme4 (version 1.1-32; Bates *et al.* 2015). VIF was determined with the function vif of the package car (version 3.0-5; Fox and Weisberg 2011).

Confidence intervals for model estimates were obtained via parametric bootstrap using the bootMer function in the lme4 package (1,000 simulations). To test fixed effects, the full model was compared to a null model without the fixed effects. To assess interaction effects, the full model was compared to a reduced model excluding the interaction term. For these model comparisons, we utilized a likelihood ratio test (Dobson 2001). If the interaction was significant, post-hoc comparisons were conducted using the emmeans package (version 1.8.5; Lenth 2023), with Bonferroni correction. Otherwise, main effects were tested by comparing the reduced model with a model that lacked the corresponding factor using a likelihood ratio test. Models were checked for outliers and influential cases using the package influence. ME (version 0.9-9; Nieuwenhuis *et al.* 2012). Model stability was assessed by iteratively removing individual participants and comparing subset-based estimates to those obtained from the full dataset. ERP components, saccade amplitude, and fixation duration were analysed using GLMMs with Gaussian error structures (lmer function); fixation duration was log-transformed. Residuals were checked for normality and homoscedasticity, with no major violations detected. The number of fixations was modelled using a Poisson GLMM, and saccade latency with a Gamma GLMM (glmer with log as link function). Dispersion parameters were subsequently checked. With a dispersion parameter of 0.336 for the Poisson model and 0.169 for the Gamma model, both were underdispersed, which may result in more conservative p-values.

The ERP models were based on 5,357 observations (participants × trials). Eye-tracking models used 5,313 observations for fixation number and duration, 4,995 for saccade amplitude, and 4,512 for saccade latency. Variations in sample size resulted from response-specific thresholds for trial exclusion.

## Results

**Figure 2.**
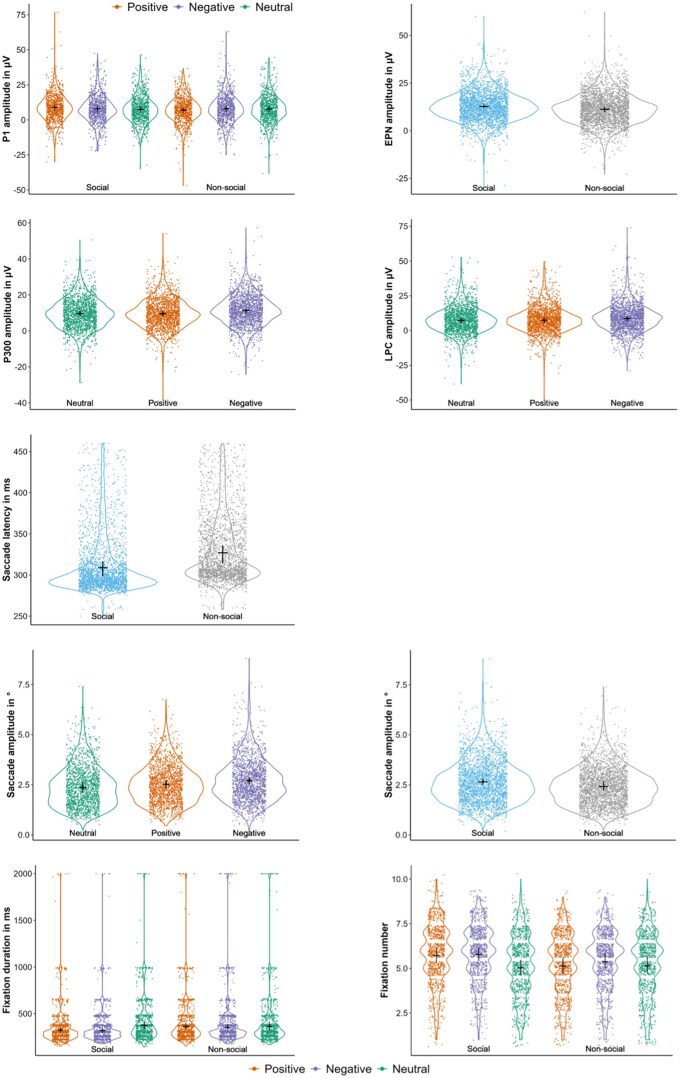
Raw data plotted together with predicted mean values and 95% confidence intervals for the respective significant interactions or main effects of social content and emotional valence on ERP and eye-tracking measures.

Model parameters are summarized in Supplementary Tables S3-S6. Observed data with predicted means and confidence intervals are visualised in Fig. 2.

### Behavioural data

Accuracy in the intact/scrambled classification task was consistently high across all conditions (all > 95%; Supplementary Table S2). For intact images, mean accuracy ranged from 97.9% (non-social positive) to 99.1% (social negative), and for scrambled images accuracy averaged 95.8%. Early responses, which triggered ‘Too early!’ feedback and were excluded from ERP and eye-tracking analyses, were rare (0.91–2.12% for intact conditions, 4.15% for scrambled; see Supplementary Table S2 for full descriptive statistics).

### ERPs

**Figure 3.**
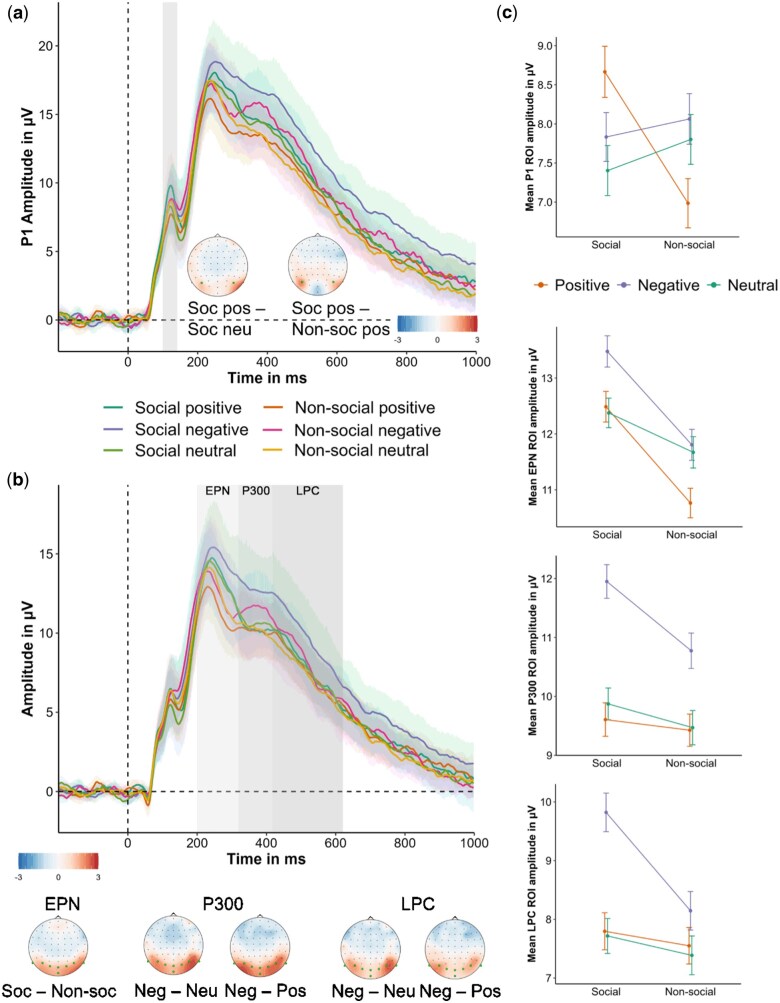
ERP amplitudes averaged across predefined regions of interest (ROIs) for each component, contrasted for conditions, with corresponding topographies for significant effects. The P1 time window is highlighted in grey (a). EPN, P300, and LPC amplitudes are shown below (b), with their time windows highlighted in different shades of grey. In the topography plots, electrodes of interest are highlighted in green. The grand mean ROI amplitudes and standard errors for each component are shown on the right (c; error bars denote ± 1 standard error of the mean).

An early interaction between social content and emotional valence was observed in **P1 amplitudes** (estimate_soc.-pos._ ± SE = –2.118 ± 0.746, estimate_soc.-neg._ ± SE = –0.253 ± 0.802, χ^2^ = 13.046, df = 2, *P* = .001; Fig. 3a, c). Social positive pictures evoked larger amplitudes than both non-social positive (estimate ± SE = 1.742 ± 0.489, *P* < .001) and social neutral images (estimate ± SE = –1.334 ± 0.536, *P* = .038). Subsequent **EPN** amplitudes showed a clear effect for social content (estimate ± SE = –1.535 ± 0.365, χ^2^ = 15.65, df = 1, *P* < .001; Fig. 3b, c), with social pictures eliciting larger amplitudes than non-social pictures. Emotional valence showed only a marginal trend (estimate_pos._ ± SE = –0.355 ± 0.472, estimate_neg._ ± SE = 0.620 ± 0.44, χ^2^ = 5.135, df = 2, *P* = .077). An effect of emotional valence was clearly reflected in later ERPs (P300: estimate_pos._ ± SE = –0.109 ± 0.457, estimate_neg._ ± SE = 1.727 ± 0.49, χ^2^ = 18.539, df = 2, *P* < .001; LPC: estimate_pos._ ± SE = 0.231 ± 0.518, estimate_neg._ ± SE = 1.415 ± 0.545, χ^2^ = 8.391, df = 2, *P* = .014; Fig. 3b, c). **P300** amplitudes were larger for negative compared to positive (estimate ± SE = –1.836 ± 0.419, *P* < .001) and neutral images (see estimate above as neutral was the reference category, *P* < .001), as were **LPC** amplitudes (negative-positive: estimate ± SE = –1.185 ± 0.464, *P* = .032; negative-neutral: estimate see above, *P* = .028). In addition, LPC amplitudes showed a trend toward a social content effect (estimate ± SE = –0.776 ± 0.424, χ^2^ = 3.112, df = 1, *P* = .078). Results for fixed effects in ERP response models are summarized in Supplementary Table S3. Complementary mass univariate analyses confirmed these component-specific effects (see Supplementary Figures S2–S4).

### Eye movement metrics

Social content and emotional valence also influenced participants’ eye movement behaviour. **Saccade latencies** (time to first saccade onset) were significantly shorter for social compared to non-social images (estimate ± SE = 0.269 ± 0.052, χ^2^ = 22.792, df = 1, *P* < .001; Fig. 1c), indicating enhanced attention capture by socially relevant scenes. Average **saccade amplitudes** were significantly larger for social compared to non-social images (estimate ± SE = –0.233 ± 0.092, χ^2^ = 6.297, df = 1, *P* = .012; Fig. 1c), and for negative compared to neutral pictures (estimate_pos._ ± SE = 0.154 ± 0.118, estimate_neg._ ± SE = 0.340 ± 0.117, χ^2^ = 8.690, df = 2, *P* = .013). Fixation patterns reflected a significant interaction between social content and emotional valence in complex visual scenes. **Number of fixations** (estimate_soc.-pos._ ± SE = –0.130 ± 0.04, estimate_soc.-neg._ ± SE = –0.096 ± 0.04, χ^2^ = 10.47, df = 2, *P* = .005; Fig. 1c) was higher for social positive compared to non-social positive (ratio ± SE = 1.113 ± 0.031, *P* < .001), for social negative compared to non-social negative (ratio ± SE = 1.076 ± 0.029, *P* = .007), and for social positive and negative compared to social neutral images (social positive-social neutral: ratio ± SE = 0.88 ± 0.025, *P* < .001, social negative-social neutral: ratio ± SE = 0.87 ± 0.027, *P* < .001). **Fixation duration** showed the exact opposite interaction pattern (estimate_soc.-pos._ ± SE = 0.144 ± 0.053, estimate_soc.-neg._ ± SE = 0.114 ± 0.054, χ^2^ = 7.401, df = 2, *P* = .025; Fig. 1c). Fixations were shorter for social positive compared to non-social positive (ratio ± SE = 0.891 ± 0.032, *P* = .001), for social negative compared to non-social negative (ratio ± SE = 0.917 ± 0.034, *P* = .019), and for social positive and negative compared to social neutral images (social positive-social neutral: ratio ± SE = 1.16 ± 0.044, *P* < .001, social negative-social neutral: ratio ± SE = 1.17 ± 0.047, *P* < .001). Results for fixed effects in eye movement response models are summarized in Supplementary Table S4.

## Discussion

This study investigated the temporal dynamics of the processing of emotional valence and social content during complex scene perception, combining ERP and eye-tracking measures. Across both neural (ERP) and behavioural (eye-tracking) measures, social content strongly influenced early and sustained attention. P1 amplitudes were modulated by a social × valence interaction, with social positive images eliciting larger responses than non-social positive and social neutral images. The EPN was enhanced for social versus non-social scenes, and later components (P300, LPC) were larger for negative images. Eye-tracking data mirrored and complemented these neural effects: first saccades were faster and average saccade amplitudes larger for social than non-social images, and amplitudes were also larger for negative than for neutral scenes. Fixation patterns showed a social × valence interaction, with both social positive and social negative images receiving more fixations of shorter duration than social neutral images. Taken together, the ERP and eye-tracking results reveal a temporally ordered sequence in which social relevance captures attention early, whereas emotional valence dominates later evaluative stages, culminating in enhanced exploration of negative content across social contexts. This integrated pattern challenges the notion of a purely valence-driven negativity bias and instead supports a relevance-based framework in which social cues modulate both early perception and later evaluation.

The **P1** effects likely reflect early attentional capture by socially salient cues rather than a fully fledged appraisal of ‘social safety’. While increased P1/P2 amplitudes are often reported for negative stimuli (e.g. Carretié *et al.* 2001), few studies have examined social content as a concurrent factor. Our results align with findings for positively conditioned faces (e.g. Hammerschmidt *et al.* 2017) and cues of safety and comfort (Beckes *et al.* 2013), and are consistent with an approach-related bias toward socially positive stimuli. Within the Component Process Model (CPM; Scherer 2001), this early stage may correspond to intrinsic pleasantness or novelty checks, with socially positive cues rapidly evaluated as goal-conducive. The absence of robust early valence effects for non-social images suggests that early negativity biases may depend on the emotional salience or personal relevance of stimuli. Non-social negative scenes in our set, although unpleasant, may have been less personally engaging than socially threatening content, reducing their capacity to elicit early bias. Future work should test this by manipulating threat potential while holding social content constant.

The **EPN** component was enhanced for social versus non-social images, consistent with our previous work (Schacht and Vrtička 2018). Its relatively anterior, P2-like distribution is in line with studies using longer viewing durations (e.g. Cuthbert *et al.* 2000; Codispoti *et al.* 2007; Thom *et al.* 2014; Lin *et al.* 2015), whereas posterior EPNs are more typical for brief presentations (e.g. Junghöfer *et al.* 2001; Schupp *et al.* 2003), suggesting that ERP morphology varies with task timing and perceptual demands. While the functional significance of the EPN remains debated, it has been linked to emotional arousal (e.g. Schupp *et al.* 2004), scenes involving joy or eroticism (Frank and Sabatinelli 2019), and human bodies (Frank and Sabatinelli 2019; Farkas *et al.* 2020; Farkas and Sabatinelli 2023). Our findings align with this interpretation, suggesting that bodily and social cues carry heightened relevance during mid-stage processing.

Later ERP components (**P300** and **LPC**) were dominated by emotional valence, with larger amplitudes for negative than positive or neutral images. These effects may reflect more elaborate evaluative or memory-related processes, particularly for threatening content, and are consistent with the well-documented sensitivity of these components to both valence and arousal. Alternatively, this pattern could also be driven by differences in arousal of negative and positive or neutral images (Olofsson *et al.* 2008; Bayer and Schacht 2014; Hajcak and Foti 2020), or by the greater task relevance and perceptual complexity often associated with negative stimuli.

Eye-tracking results converged with the ERP findings, indicating rapid and sustained prioritization of socially and emotionally relevant content. First saccades were faster for social compared with non-social images, consistent with reflexive attention capture (End and Gamer 2017; Rösler *et al.* 2017; Flechsenhar *et al.* 2018). While the P1 peaked around 100–140 ms, these first saccades occurred later (304 ms for social and 392 ms for non-social images), in consistent with the notion that covert attention precedes overt gaze shifts (Calvo and Lang 2005; Carrasco 2011; Simola *et al.* 2013). Subsequent viewing behavior revealed that both social positive and social negative images received more intense exploration than social neutral scenes, as indicated by a higher number of shorter fixations. Saccade amplitudes were also larger for social than for non-social images and for negative than for neutral images, the latter possibly reflecting broader attentional scanning linked to threat detection. These metrics—particularly increased fixation counts and larger saccade amplitudes—provide behavioral evidence for active information seeking in response to biologically and motivationally relevant cues (Bradley 2009; Bradley *et al.* 2011). Prior work has shown more fixations to negative images (Humphrey *et al.* 2012; Niu *et al.* 2012), and stronger gaze allocation to social versus physically salient features (Birmingham *et al.* 2009; End and Gamer 2017; Rösler *et al.* 2017). Our findings extend this evidence by demonstrating that emotional modulations of attention occur even within complex social scenes.

Integrating across modalities, both ERP and eye-tracking data indicate an early prioritization of social content followed by enhanced exploration of emotionally salient scenes. The correspondence between P1 amplitudes and saccade latency suggests a relevance-driven mechanism rather than a purely valence-driven one: the ERP effect was strongest for socially positive images, whereas eye-tracking revealed faster first saccades to social content regardless of valence. Emotionally salient social scenes—both positive and negative—elicited more and shorter fixations, indicating sustained exploration. Across contexts, negative content was associated with larger saccade amplitudes, consistent with broader attentional scanning and potential threat-detection mechanisms. Emotional valence exerted stronger influence at later ERP stages (P300, LPC) and in sustained gaze patterns, highlighting that while both measures are sensitive to relevance, they capture different aspects of valence-related processes.

Together, our findings indicate that the instinctive threat-avoidance mechanism—often proposed to underlie the negativity bias—can be attenuated or even overridden by an initial approach response rooted in the inherent relevance of socially positive cues. Within the framework of appraisal theories, this pattern is consistent with an early evaluation of intrinsic pleasantness and goal conduciveness, particularly in contexts where social cues signal safety despite the presence of potentially deterrent stimuli. We defined social content as the presence of one or more humans, in line with prior work (Vrtička *et al.* 2011, 2012, 2013; End and Gamer 2017; Rösler *et al.* 2017; Flechsenhar *et al.* 2018; Schacht and Vrtička 2018; Tso *et al.* 2018; Weierich *et al.* 2019). However, the specific cues that distinguish social from non-social content remain insufficiently specified. Recent work highlights the importance of a more detailed taxonomy of visual features—including the number of people, gaze direction, face visibility, and interaction cues (e.g. McMahon and Isik 2023; Santavirta *et al.* 2024) – to better understand how socio-emotional relevance is computed. This issue is addressed more systematically in a recent rating study (Mitschke *et al.* 2025), in which a large set of affective scenes, including many used here, was coded for objective social features and evaluated on multiple socio-emotional dimensions.

Building on this, a key limitation of our study is the use of static images, which constrains ecological validity and limits generalizability to dynamic, real-world social interactions. Moreover, the absence of a fine-grained taxonomy of visual scene features restricts interpretability: without systematic coding of interpersonal cues, emotional expressions, body language, or goal-directed actions, it remains unclear which specific socio-emotional elements drive the observed effects beyond the broad ‘social’ versus ‘non-social’ distinction. This limitation is compounded by the use of an all-female sample, which restricts generalizability and may mask potential sex differences in socio-emotional processing. In addition, there was a modest imbalance in the number of individuals and faces across conditions, and the non-social category may differ not only in human presence but also in the absence of implied interpersonal context, potentially introducing interpretational confounds. Further, although our GLMM approach with random effects for participants and stimuli improves internal validity, it does not address these external validity constraints. Finally, while our main analyses used a shared posterior ROI for the EPN, P300, and LPC to ensure comparability with prior work, this approach may obscure topographic differences between components. Complementary mass univariate analyses (see Supplementary Figures S2–S4) confirmed component-specific scalp distributions, but we note this limitation for future ERP studies.

## Conclusion

This study demonstrates that social and emotional content jointly guide visual attention to complex scenes, exerting both independent and interactive effects in a temporally ordered sequence. The findings align with appraisal theories proposing a cascade of relevance checks, in which social content is evaluated before emotional valence, and they challenge the notion of a universal negativity bias in socially rich contexts. Future research—using more diverse participant samples and stimuli coded for fine-grained socio-emotional features—is needed to clarify which specific cues drive these effects and to test their generalizability to dynamic, real-world interactions.

## Supplementary Material

nsaf108_Supplementary_Data

## Data Availability

Pre-processed EEG and eye-tracking data are available in the Open Science Framework, at https://osf.io/7fb86/; analysis code is available upon request.

## Supplementary Material

See the online supplementary material for detailed stimulus validation procedures, additional analyses, and figures.
