# Supplementary material for: Challenging the negativity bias in affective scene viewing: The role of social content
Source: Soc Cogn Affect Neurosci. 2025 Oct 14;20(1):nsaf108. doi: 10.1093/scan/nsaf108 (PMC12706866; doi:10.1093/scan/nsaf108)
Supplement: nsaf108_Supplementary_Data [file nsaf108_supplementary_data.docx]

**Supplementary material_ SCAN-25-R168.R2**

**Stimulus validation**

The subset of images used in this study was again validated by entering the mean ratings of pleasantness and arousal (averaged across raters for each image) into a two (social content: social, non-social) × three (valence category: positive, negative, neutral) between-groups analysis of variance (ANOVA) at the image level. No main effect of social content was found for ratings of pleasantness (F_(1,39)_ = 0.014, p = .907) and intensity (F_(1,39)_ = 0.359, p = .553). No interaction between social content and valence category was found for either rating category (F_(1,39)_ = 0.506, p = .481, and, F_(1,39)_ = 0.003, p = .957, respectively). However, main effects of valence category were found (F_(1,39)_ = 3958.49, p < .001 and F_(1,39)_ = 1383.408, p < .001), which arose from significant positive > neutral > negative differences in valence ratings and significant negative > positive > neutral differences in arousal ratings. These valence effects were expected and resulted from positive content being judged as more pleasant than neutral or negative content and emotional content being judged as more intense than emotionally neutral content. In addition, all images were matched for their low-level properties (contrast, luminance, and spatial frequency). No significant main effects of social content or valence category and no interaction effects were detected for contrast (F_(1,39)_ < 0.018, p > .676) and luminance (F_(1,39)_ < 1.33, p > .277). For spatial frequency, (Haar) wavelet coefficients representing the energy at a given frequency were extracted from nine frequency bands for the grayscale version of each image. The respective values for the two highest and lowest frequency bands were averaged and validated with a 2 x 3 rmANOVA. No significant main or interaction effects were found in the high (F_(1,39)_ < 2.209, p > .124) and low (F_(1,39)_ < 0.792, p > .379) frequency bands.

**Control analyses: image complexity and physical saliency**

To ensure our findings were not driven by stimulus-related confounds, we conducted additional checks for image complexity and physical salience. Image complexity was quantified using root-mean-square error (RMSE) between each grayscale original image and its grayscale gif-compressed version, divided by the compression ratio, defined as the quotient of the uncompressed to compressed grayscale image file size (Yu and Winkler, 2013; Durmus, 2020). Because the number of images varied across conditions (40 in each positive/negative, 20 in each neutral), we randomly sampled 20 images per condition 10,000 times. For each iteration, we ran between-subject ANOVAs with social content and emotional valence as factors to test for condition-specific complexity differences. Homogeneity of variance and normality of residuals were checked with the package performance (version 0.10.2; Lüdecke *et al.*, 2021). No violations were detected. Only 38 out of the 10,000 ANOVAs yielded a significant result (p < .05) for an interaction effect (0.38 %), 182 for a main effect of valence (1.82 %), and 8 for a main effect of social content (0.08 %). These results indicate that the presence of significant effects for complexity differences across conditions was relatively small, suggesting that both factors may not have a substantial impact on the observed complexity variations.

To examine spatial clustering of physically salient image regions between conditions, we computed graph-based visual saliency using the Spectral Visual Saliency Toolbox (version 1.20.0.0) in Matlab (R2018a, MathWorks Inc.). Pixel-wise saliency values were averaged across images per condition and visualized as heatmaps, with colour scales anchored to the mean, minimum, and maximum across all conditions. These heatmaps do not show any obvious clustering in the centre region in one condition compared to the others (see Supplementary Figure S1).


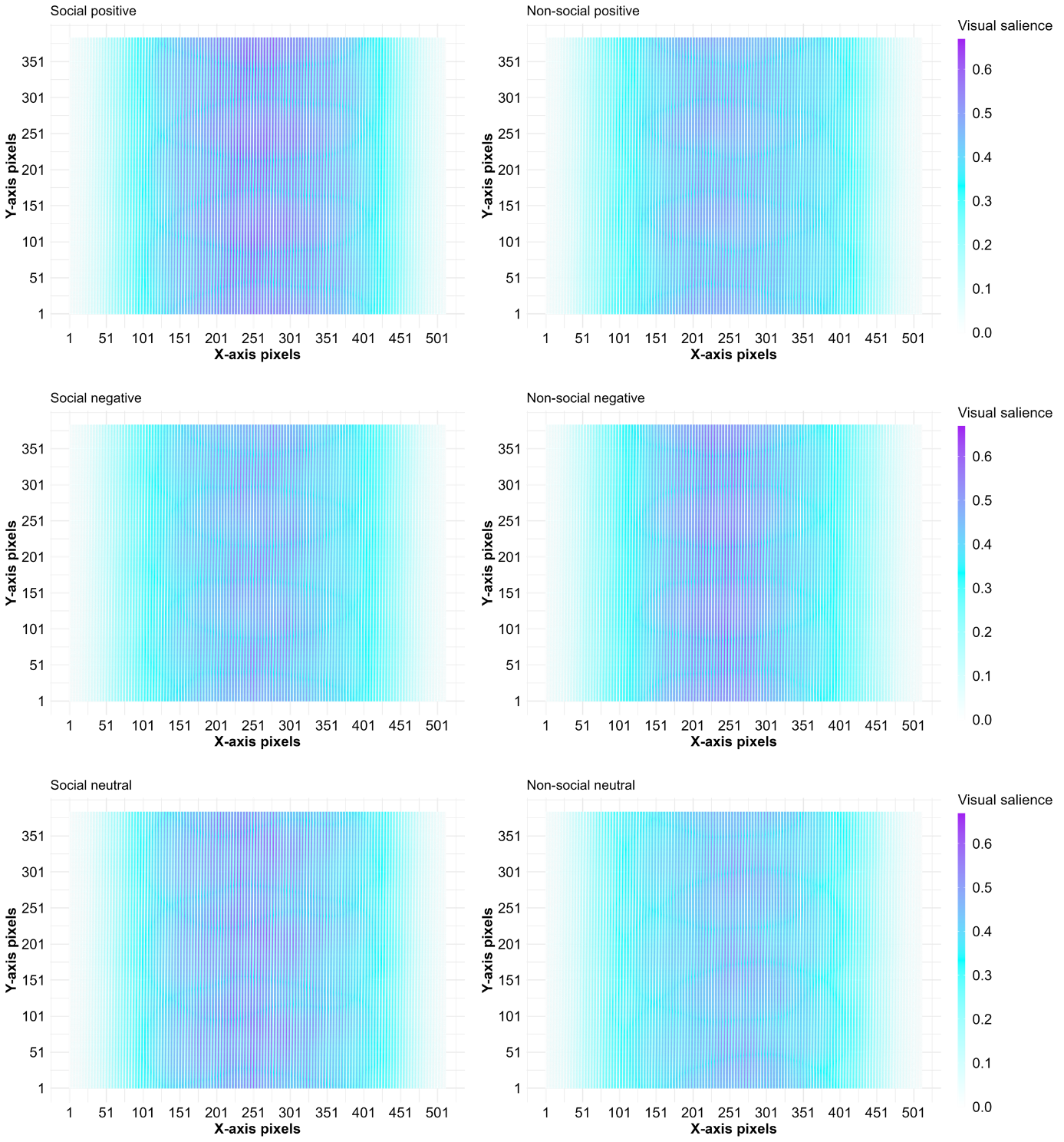


**Figure S1.** Averaged Visual salience values per pixel (512 x 384) plotted as heatmaps for each condition. The colour scales are anchored to the mean, the minimum, and maximum of the range of all conditions.

**Relevance detection follow-up**

In addition, social content and emotional valence may influence relevance detection during natural viewing (Niu *et al.*, 2012), potentially affecting both eye movements and ERPs. To examine whether relevant image regions were systematically biased in location across conditions, we conducted a follow-up study. All 200 images were presented to 24 healthy female participants who were asked to freely indicate the regions that were relevant to them on a computer screen by mouse click within the six seconds of image presentation. We then counted the number of clicks falling inside and outside the foveal region, defined as a circular area with a diameter of 73 pixels (corresponding to 2° of visual angle) centred on the fixation cross (see Apparatus). A GLMM with a binomial error structure and a logit link function was fitted to the data using the function glmer. With a dispersion parameter of 0.619, the model was slightly underdispersed. The full-null model comparison did not yield a statistically significant result (full-null comparison: χ^2^ = 27.907, df = 20, p = .112). This suggests that the inclusion of both predictor variables in the full model did not significantly improve model fit compared to the null model, indicating that social content and emotional valence may not have had a significant impact on relevance detection inside and outside the fovea.

**Mass univariate analysis**

The choice of electrodes and time windows for the analyses reported in the main manuscript was based on the article by Schacht and Vrtička (2018). However, the selection of electrode clusters and time windows for EPN, P300, and LPC vary across the literature. To complement our predefined regions of interest (ROIs), we conducted a mass univariate analysis (MUA) with permutation-based correction for multiple comparisons (Groppe *et al.*, 2011), providing a more data-driven view of spatial and temporal distribution of our effects.

Because the mass univariate approach benefits in terms of statistical power when using predefined time windows and spatial ROIs (Fields and Kuperberg, 2020), a broad spatiotemporal ROI was established for the EPN, P300, and LPC. The ROI included posterior electrodes, excluding frontal channels, and a time window from 150-700 ms. The analysis was implemented using MATLAB (R2018a) with EEGLAB (version 2021.1), the Mass Univariate ERP toolbox (Groppe *et al.*, 2011), and the Factorial Mass Univariate ERP toolbox (Fields, 2017). First, an ANOVA was computed with the factors valence, social content, and their interaction at each electrode and time point within the predefined ROI. Spatial and temporal clusters of significant effects were identified by grouping adjacent electrodes and time points that exceeded a predefined threshold (p < 0.01). Cluster mass was calculated as the sum of F-values across electrodes and time points in each cluster. A permutation test was used to control for type I error due to multiple comparisons. Condition labels for ERP data were randomly reassigned within each participant and cluster masses were recalculated for 50,000 permutations. A cluster was considered statistically significant if its observed mass was within the top 5 % (family-wise α level of 0.05) of the permuted distribution.

*Results*

The mass univariate analysis revealed a significant interaction of social x emotional content in two clusters (see Supplementary Figure S2). Cluster one comprised electrodes TP7, CP6, P3, P7, P9, P4, P6, P8, P10, PO3, PO7, PO4, PO8, Oz, O1, Iz between 150-574 ms (p = .035; spatial peak at P10 and temporal peak at 152 ms). Cluster two comprised electrodes Cz, C1, C2, C4, CPz, CP1, CP3, CP4, CP6, P1 between 296-700 ms (p = .010; spatial peak at C4 and temporal peak at 332 ms).

Regarding the effect of social content (see Supplementary Figure S3), one cluster was found including electrodes Cz, C1, C3, C5, T7, C2, C4, C6, CPz, CP1, CP3, TP7, CP2, CP4, CP6, TP8, Pz, P1, P3, P5, P7, P9, P2, P4, P6, P8, P10, POz, PO3, PO7, PO4, PO8, Oz, O1, O2, Iz between 150-700 ms (p = .001; spatial peak at O2 and temporal peak at 278 ms). For emotional content (see Supplementary Figure S4) one cluster emerged comprising electrodes Cz, C1, C3, C5, C2, C4, T8, CPz, CP1, CP3, CP5, CP2, CP4, CP6, TP8, Pz, P1, P3, P5, P7, P9, P2, P6, P8, P10, POz, PO3, PO7, PO4, PO8, Oz, O1, O2, Iz between 150-700 ms (p < .001; spatial peak at PO8 and temporal peak at 374 ms).

The MUA results revealed an interaction in both a parietooccipital and a centroparietal cluster. While the effect of social content started in parietooccipital regions and shifted to central regions in the later time window, emotional content showed the opposite pattern, starting in the entire posterior ROI and persisting only in parietooccipital electrodes in the later stages. This suggests that effects extended beyond the ROI defined in the main analysis to central electrodes. However, when comparing the results of the MUA with the results of the GLMMs reported above, it should be noted that the mass univariate analysis is based on repeated measures ANOVAs. These include random intercept effects for subject ID, but not the random slope structure, nor the random intercept effect for picture ID. This may have contributed to the divergent results between the two statistical methods.


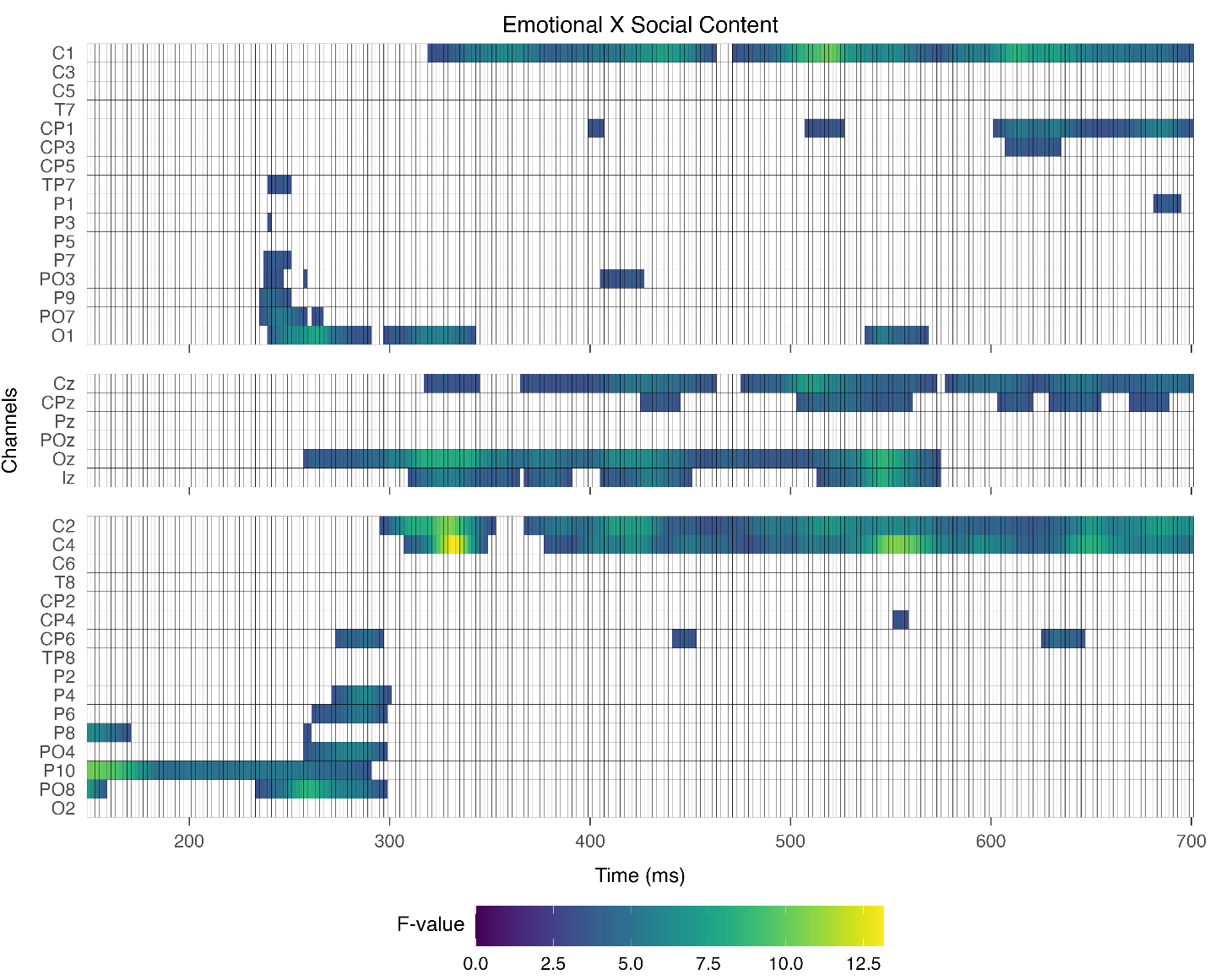


**Figure S2.** Results of mass univariate analysis for the interaction of Social Content and Emotional Content/valence, with electrodes on the y-axis and Time points in ms on the x-axis. Only F-values of clusters that survived permutation tests to control for type I error due to multiple comparisons are shown.


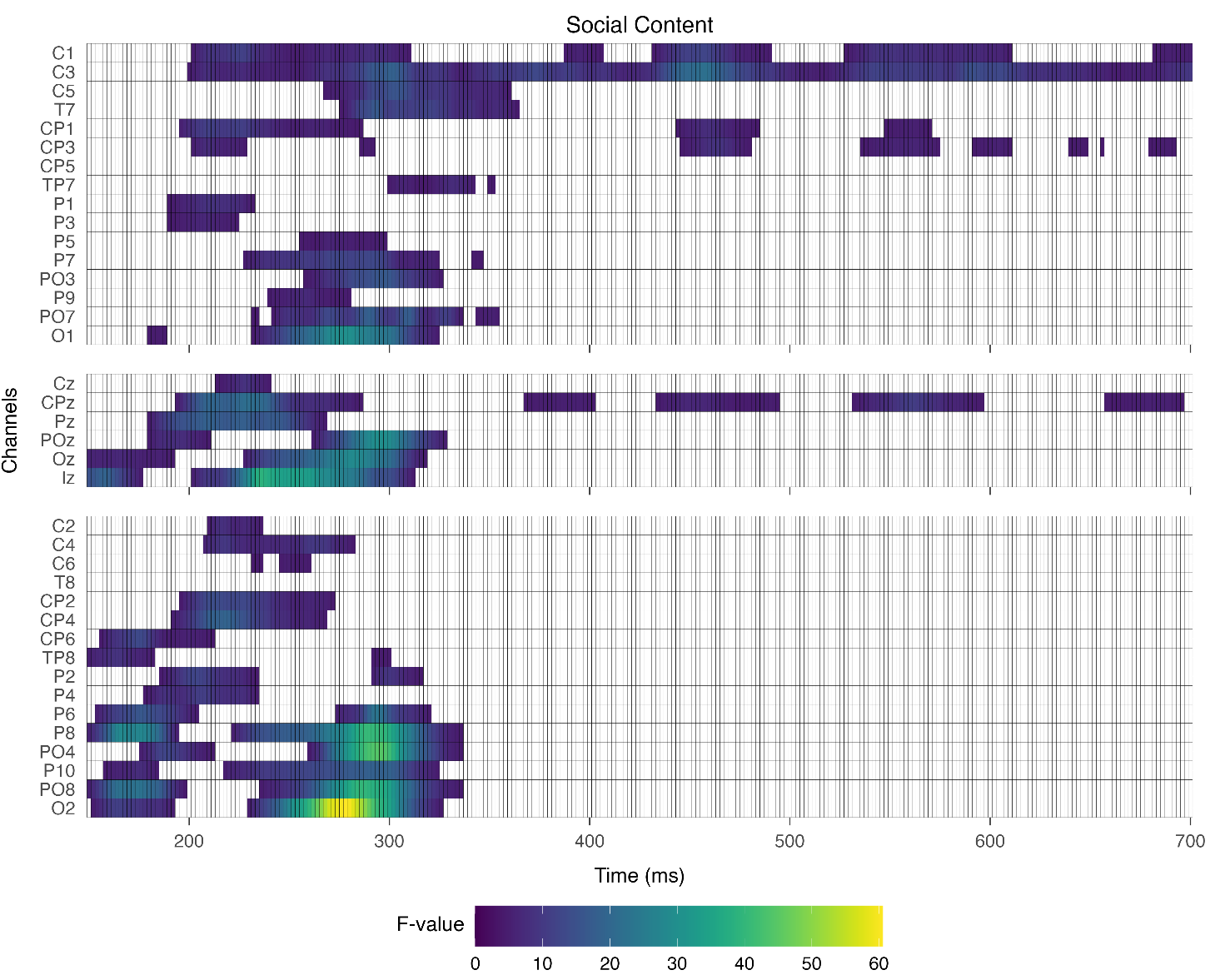


**Figure S3.** Results of mass univariate analysis for the effect of Social Content, with electrodes on the y-axis and Time points in ms on the x-axis. Only F-values of clusters that survived permutation tests to control for type I error due to multiple comparisons are shown.


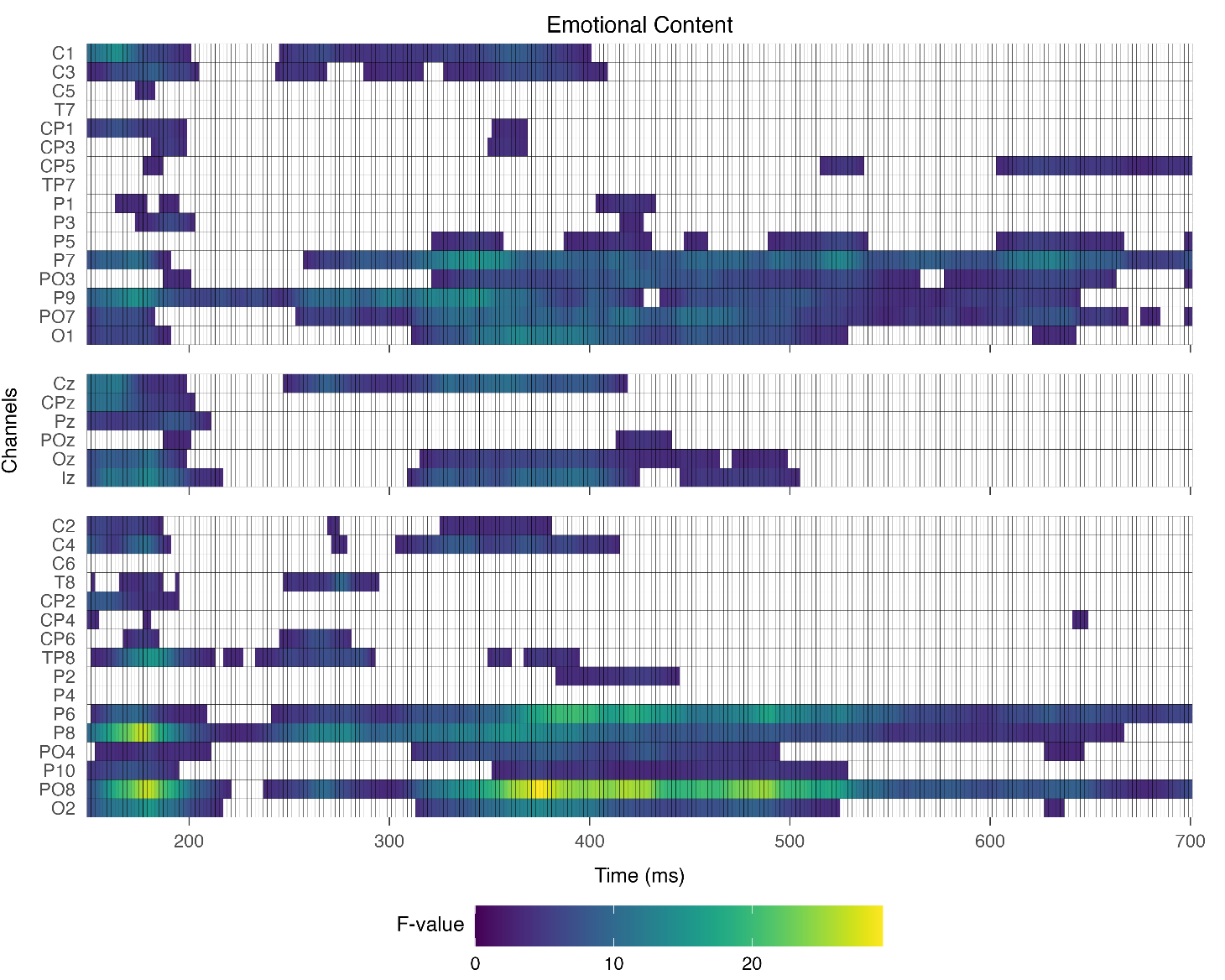


**Figure S4.** Results of mass univariate analysis for the effect of Emotional Content/valence, with electrodes on the y-axis and Time points in ms on the x-axis. Only F-values of clusters that survived permutation tests to control for type I error due to multiple comparisons are shown.

**Table S1.** Number of pictures with social content depicting the respective number of people and visible faces for each valence category.

| picture content | N positive | N negative | N neutral |
| --- | --- | --- | --- |
| number of people |  |  |  |
| 1 | 3 | 16 | 17 |
| 2 | 19 | 10 | 3 |
| 3 | 10 | 2 | 0 |
| 4 | 3 | 4 | 0 |
| 5 | 3 | 5 | 0 |
| 6 | 2 | 3 | 0 |
| number of faces |  |  |  |
| 1 | 7 | 19 | 16* |
| 2 | 19 | 6 | 2 |
| 3 | 7 | 2 | 0 |
| 4 | 4 | 2 | 0 |
| 5 | 1 | 2 | 0 |
| 6 | 2 | 2 | 0 |
| faces not visible | 0 | 7 | 2 |

* The neutral category included four close-up portraits in front of a neutral background, while all other pictures with social content showed human figures in more complex scenes.

**Table S2.** Accuracy and early-response rates for intact/scrambled classification task by condition

| **Condition** | **Accuracy (%) M ± SD** | **Early responses (%) M ± SD** |
| --- | --- | --- |
| Non-social positive (intact) | 97.9 ± 4.47 | 2.12 ± 1.13 |
| Social positive (intact) | 98.8 ± 5.88 | 1.25 ± 0.52 |
| Non-social negative (intact) | 99.0 ± 5.97 | 1.03 ± 0.35 |
| Social negative (intact) | 99.1 ± 6.06 | 0.91 ± 0.89 |
| Non-social neutral (intact) | 98.0 ± 5.59 | 2.02 ± 1.03 |
| Social neutral (intact) | 98.2 ± 8.18 | 1.82 ± 0.65 |
| Scrambled | 95.8 ± 4.80 | 4.15 ± 1.62 |

*Note. Accuracy values reflect the percentage of correct intact/scrambled classifications; early responses are trials in which participants responded before the response cue and received “Too early!” feedback. Both measures are reported as means and standard deviations across participants.*

**Table S3.** Results for fixed effects in ERP response models. Shown are estimates and standard errors, together with confidence intervals, results of tests, and the range of estimates obtained when dropping levels of random effects one at a time.

| term | estimate | SE | lower CI | upper CI | t | p | min | max |
| --- | --- | --- | --- | --- | --- | --- | --- | --- |
| P1 | |  |  |  |  |  |  |  |
| intercept | 7.402 | 1.109 | 5.280 | 9.534 | 6.677 |  | 6.712 | 7.723 |
| social | 0.376 | 0.599 | -0.836 | 1.535 | 0.627 |  | 0.109 | 0.610 |
| valence |  |  |  |  |  |  |  |  |
| positive | 1.334 | 0.536 | 0.317 | 2.420 | 2.491 |  | 1.069 | 1.539 |
| negative | 0.479 | 0.571 | -0.586 | 1.632 | 0.838 |  | 0.213 | 0.704 |
| interaction |  |  |  |  |  | .001 |  |  |
| social:pos | -2.118 | 0.746 | -3.633 | -0.635 | -2.838 |  | -2.353 | -1.852 |
| social:neg | -0.253 | 0.802 | -1.801 | 1.280 | -0.315 |  | -0.593 | 0.014 |
| Full-null comparison: χ^2^ = 13.556, df = 5, p = .019  Full-reduced comparison: χ^2^ = 13.046, df = 2, p = .001 | | | | | | | | |
| EPN^1^ | |  |  |  |  |  |  |  |
| intercept | 12.714 | 0.796 | 11.177 | 14.277 | 15.968 |  | 12.377 | 13.028 |
| social | -1.535 | 0.365 | -2.223 | -0.823 | -4.209 | .000 | -1.623 | -1.424 |
| valence |  |  |  |  |  | .077 |  |  |
| positive | -0.355 | 0.472 | -1.276 | 0.509 | -0.752 |  | -0.488 | -0.209 |
| negative | 0.620 | 0.440 | -0.236 | 1.487 | 1.410 |  | 0.487 | 0.720 |
| interaction |  |  |  |  |  | .454 |  |  |
| Full-null comparison: χ^2^ = 20.371, df = 5, p = .001  Full-reduced comparison: χ^2^ = 1.578, df = 2, p = .454 | | | | | | | | |
| P300^1^ | |  |  |  |  |  |  |  |
| intercept | 9.935 | 0.695 | 8.583 | 11.291 | 14.295 |  | 9.691 | 10.158 |
| social | -0.651 | 0.387 | -1.380 | 0.181 | -1.682 | .099 | -0.830 | -0.532 |
| valence |  |  |  |  |  | .000 |  |  |
| positive | -0.109 | 0.457 | -1.016 | 0.758 | -0.240 |  | -0.255 | 0.012 |
| negative | 1.727 | 0.490 | 0.745 | 2.724 | 3.523 |  | 1.596 | 1.886 |
| interaction |  |  |  |  |  | .46 |  |  |
| Full-null comparison: χ^2^ = 22.401, df = 5, p < .001  Full-reduced comparison: χ^2^ = 1.552, df = 2, p = .46 | | | | | | | | |
| LPC | |  |  |  |  |  |  |  |
| intercept | 7.655 | 0.905 | 5.926 | 9.537 | 8.454 |  | 7.260 | 7.892 |
| social | -0.776 | 0.424 | -1.657 | 0.039 | -1.832 | .078 | -0.883 | -0.635 |
| valence |  |  |  |  |  | .014 |  |  |
| positive | 0.231 | 0.518 | -0.805 | 1.197 | 0.446 |  | 0.097 | 0.368 |
| negative | 1.415 | 0.545 | 0.393 | 2.499 | 2.597 |  | 1.222 | 1.557 |
| interaction |  |  |  |  |  | .299 |  |  |
| Full-null comparison: χ^2^ = 14.260, df = 5, p = .014  Full-reduced comparison: χ^2^ = 2.415, df = 2, p = .299 | | | | | | | | |

Factor social was manually dummy coded and then mean centred for the random effects part.

Factor valence was manually dummy coded with neutral being the reference category and then mean centred for the random effects part.

P-values for main effects are omitted when a significant interaction is present, as these main effects cannot be meaningfully interpreted in isolation (see Methods). For P1, the significant social × valence interaction precludes independent interpretation of the main effects.

^1^ One influential case was excluded. Estimates of main effects for full sample EPN: social = -1.605 (CI lower = -2.368, CI upper = -0.907); P300: positive = -0.210 (CI lower = -1.100, CI upper = 0.663), negative = 1.722 (CI lower = 0.766, CI upper = 2.671).

**Table S4.** Results for fixed effects in eye movement response models. Shown are estimates and standard errors, together with confidence intervals, results of tests, and the range of estimates obtained when dropping levels of random effects one at a time.

| term | estimate | SE | lower CI | upper CI | t | p | min | max |
| --- | --- | --- | --- | --- | --- | --- | --- | --- |
| Saccade latency | |  |  |  |  |  |  |  |
| intercept | -1.253 | 0.086 | -1.507 | -1.069 | -14.491 |  | -1.279 | -1.233 |
| social | 0.269 | 0.052 | 0.113 | 0.432 | 5.192 | .000 | 0.261 | 0.281 |
| valence |  |  |  |  |  | .871 |  |  |
| positive | 0.023 | 0.060 | -0.184 | 0.202 | 0.386 |  | 0.015 | 0.038 |
| negative | 0.065 | 0.066 | -0.150 | 0.253 | 0.996 |  | 0.057 | 0.080 |
| interaction |  |  |  |  |  | .303 |  |  |
| Full-null comparison: χ^2^ = 25.027, df = 5, p < .001  Full-reduced comparison: χ^2^ = 2.387, df = 2, p = .303 | | | | | | | | |
| Saccade amplitude | | | |  |  |  |  |  |
| intercept | 2.481 | 0.128 | 2.240 | 2.723 | 19.336 |  | 2.422 | 2.516 |
| social | -0.233 | 0.092 | -0.409 | -0.048 | -2.532 | .012 | -0.249 | -0.207 |
| valence |  |  |  |  |  | .013 |  |  |
| positive | 0.154 | 0.118 | -0.066 | 0.395 | 1.301 |  | 0.124 | 0.204 |
| negative | 0.339 | 0.117 | 0.116 | 0.577 | 2.885 |  | 0.307 | 0.386 |
| interaction |  |  |  |  |  | .555 |  |  |
| Full-null comparison: χ^2^ = 15.452, df = 5, p = .009  Full-reduced comparison: χ^2^ = 1.168, df = 2, p = .558 | | | | | | | | |
| Fixation number | | | | |  |  |  |  |
| intercept | 1.615 | 0.044 | 1.532 | 1.7 | 36.985 |  | 1.601 | 1.641 |
| social | 0.023 | 0.031 | -0.036 | 0.080 | 0.741 |  | 0.008 | 0.036 |
| valence |  |  |  |  |  |  |  |  |
| positive | 0.127 | 0.029 | 0.073 | 0.183 | 4.450 |  | 0.113 | 0.140 |
| negative | 0.140 | 0.030 | 0.081 | 0.199 | 4.588 |  | 0.125 | 0.153 |
| interaction |  |  |  |  |  | .005 |  |  |
| social:pos | -0.130 | 0.040 | -0.204 | -0.055 | -3.219 |  | -0.143 | -0.115 |
| social:neg | -0.096 | 0.040 | -0.176 | -0.019 | -2.387 |  | -0.109 | -0.081 |
| Full-null comparison: χ^2^ = 28.635, df = 5, p < .001  Full-reduced comparison: χ^2^ = 10.47, df = 2, p = .005 | | | | | | | | |
| Fixation duration | | | | |  |  |  |  |
| intercept | -0.982 | 0.056 | -1.095 | -0.874 | -17.414 |  | -1.011 | -0.966 |
| social | -0.028 | 0.044 |  |  | -0.630 |  | -0.043 | -0.006 |
| valence |  |  |  |  |  |  |  |  |
| positive | -0.144 | 0.038 | -0.218 | -0.073 | -3.799 |  | -0.159 | -0.122 |
| negative | -0.154 | 0.041 | -0.229 | -0.077 | -3.783 |  | -0.168 | -0.131 |
| interaction |  |  |  |  |  | .025 |  |  |
| social:pos | 0.144 | 0.053 | 0.043 | 0.247 | 2.699 |  | 0121 | 0.159 |
| social:neg | 0.114 | 0.054 | 0.007 | 0.219 | 2.126 |  | 0.092 | 0.130 |
| Full-null comparison: χ^2^ = 22.034, df = 5, p < .001  Full-reduced comparison: χ^2^ = 7.401, df = 2, p = .025 | | | | | | | | |

Factor social was manually dummy coded and then mean centred for the random effects part.

Factor valence was manually dummy coded with neutral being the reference category and then mean centred for the random effects part.

P-values for main effects are reported only when no significant interaction is present. When an interaction is significant, the main effects are not meaningful in isolation and are therefore omitted (consistent with Table S2 and the analysis approach described in the Methods).

**Table S5.** Estimated standard deviations for the contribution of the random effects in ERP response models.

| term | effect | sd (P1) | sd (EPN) | sd (P300) | sd (LPC) |
| --- | --- | --- | --- | --- | --- |
| image | intercept | 1.209 | 1.831 | 1.834 | 2.239 |
| participant | intercept | 5.117 | 3.557 | 2.954 | 4.224 |
|  | social | 0.474 | 0.821 | 0.900 | 0.747 |
|  | valence pos. | 0.816 | 0.986 | 0.632 | 0.351 |
|  | valence neg. | 0.655 | 0.542 | 1.064 | 0.912 |
|  | social:val.pos. | 1.187 | 0.390 | 1.447 | 1.856 |
|  | social:val.neg. | 1.878 | 0.473 | 1.718 | 2.551 |
| residual | - | 7.875 | 6.853 | 7.500 | 8.171 |

Intercept denotes random intercepts, other entries random slopes effects.

**Table S6.** Estimated standard deviations for the contribution of the random effects in eye movement response models.

| term | effect | sd (SacLat) | sd (SacAmp) | sd (FixNum) | sd (FixDur) |
| --- | --- | --- | --- | --- | --- |
| image | intercept | 0.169 | 0.586 | 0.070 | 0.128 |
| participant | intercept | 0.116 | 0.404 | 0.172 | 0.217 |
|  | social | 0.071 | 0.157 | 0.037 | 0.076 |
|  | valence pos. | 0.022 | 0.111 | 0.017 | 0.026 |
|  | valence neg. | 0.070 | 0.088 | 0.046 | 0.073 |
|  | social:val.pos. | 0.092 | 0.194 | 0.024 | 0.025 |
|  | social:val.neg. | 0.123 | 0.161 | 0.025 | 0.049 |
| residual | - | 0.425 | 0.694 | - | 0.321 |

Intercept denotes random intercepts, other entries random slopes effects.
